# Detection of miR-155 Using Peptide Nucleic Acid at Physiological-like Conditions by Surface Plasmon Resonance and Bio-Field Effect Transistor

**DOI:** 10.3390/bios14020079

**Published:** 2024-02-01

**Authors:** Francesco Lavecchia di Tocco, Valentina Botti, Salvatore Cannistraro, Anna Rita Bizzarri

**Affiliations:** Biophysics and Nanoscience Centre, DEB, Università della Tuscia, Largo dell’Università, 01100 Viterbo, Italy; francesco.ditocco@unitus.it (F.L.d.T.); valentina.botti@unitus.it (V.B.); cannistr@unitus.it (S.C.)

**Keywords:** biosensors, microRNA, surface plasmon resonance, field effect transistors

## Abstract

MicroRNAs are small ribonucleotides that act as key gene regulators. Their altered expression is often associated with the onset and progression of several human diseases, including cancer. Given their potential use as biomarkers, there is a need to find detection methods for microRNAs suitable for use in clinical setting. Field-effect-transistor-based biosensors (bioFETs) appear to be valid tools to detect microRNAs, since they may reliably quantitate the specific binding between the immobilized probe and free target in solution through an easily detectable electrical signal. We have investigated the detection of human microRNA 155 (miR-155) using an innovative capturing probe constituted by a synthetic peptide nucleic acid (PNA), which has the advantage to form a duplex even at ionic strengths approaching the physiological conditions. With the aim to develop an optimized BioFET setup, the interaction kinetics between miR-155 and the chosen PNA was preliminarily investigated by using surface plasmon resonance (SPR). By exploiting both these results and our custom-made bioFET system, we were able to attain a low-cost, real-time, label-free and highly specific detection of miR-155 in the nano-molar range.

## 1. Introduction

**Figure 1:**
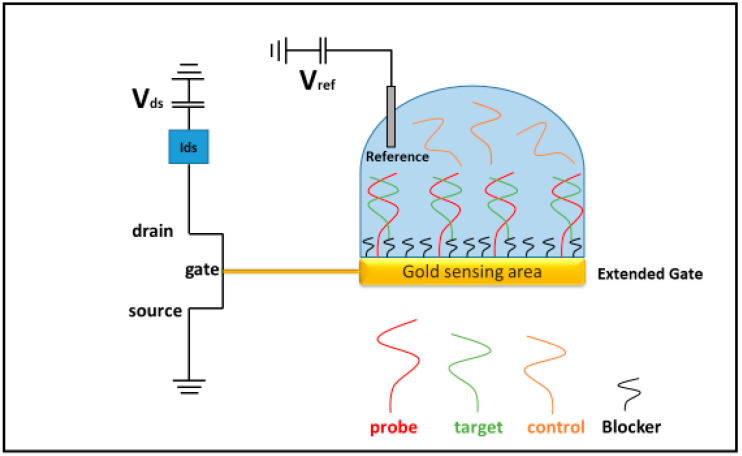
Schematic of a bioFET setup for the detection of microRNAs.

MicroRNAs (miRNAs) are small non-coding nucleic acids of approximately 18–25 nucleotides that play a pivotal role in the gene regulation of all superior organisms, including humans, and are involved in several cellular processes, such as proliferation, differentiation and apoptosis [1,2,3]. Given their physiological importance, the deregulation of miRNAs is related to the development of numerous disease states, including multiple forms of cancer [4,5] Therefore, miRNAs are excellent molecular biomarkers with high potentialities in diagnostics [6,7,8]. However, their small size and low concentrations make the detection of miRNAs rather difficult. Typical levels of circulating miRNAs in serum have been estimated in the 0.2 fM to 20 pM range and several studies suggest that their concentrations in the presence of a pathological state could increase more than 1000 times [9,10,11]; however, a precise characterization of microRNA levels is lacking, and moreover, different microRNAs can be dysregulated to varying degrees depending on the underlying pathology [12]. Currently, oligonucleotides are essentially detected by biomolecular techniques (e.g., real time PCR, Northern blotting, microarray analysis) [13,14,15]. These methods, which represent the golden standard techniques, are well-known and may reach relatively high performances with a limit of detection (LOD) in the femto-aptomolar range [16,17]. However, they require long waiting times, necessitate the use of specific labels and often involve extensive sample handling by highly specialized personnel with significant costs [18]. Biosensor technologies are susceptible to offer good solutions to the above-mentioned problems, allowing, additionally, optimized and sensitive clinical detection of miRNAs [19,20]. A biosensor is a device able to perceive biological events (e.g., the interaction between two molecular partners) and to transduce them in the form of a chemical, physical or electrical signal [21]. In the recent decades, electrochemical biosensors, and particularly bio-field effect transistor (bioFET)-based devices, have received great attention, essentially because they can reach levels of sensitivity and specificity comparable to the canonical methods, being at the same time label-free, low cost and presenting high potentiality to be automatized with no or little sample pre-treatment [22,23,24,25]. A schematic representation of a bioFET architecture for microRNA detection is depicted in Figure 1.

Briefly, in a FET device, the electric current flows between two electrodes (source and drain) linked by a semiconductor channel; a third electrode (gate), coupled to the device through a thin dielectric layer, can modulate the conductance between the drain–source electrodes according to its voltage [26]. In the extended gate configuration, an external electrode is connected to the gate to avoid a direct interaction between the transistor gate and the biological solution. When specific biological probes, previously immobilized onto the sensor surface, capture the target of interest, a release of charges towards the gate electrode occurs. The change of the gate voltage then affects the source–drain current, whose variation can be put into relationship to the target concentration [27]. However, current detection involves only the accumulated charges within the so-called Debye distance from the electrode, which, in turn, strongly depends on the ionic strength of the solution [28,29]. Natural capturing probes for miRNAs are constituted by their complementary DNA or RNA strands, which give rise to duplexes via a hybridization process. Since both partners display negatively charged phosphate backbones, a high ionic strength is required to optimize the hybridization process; approximately 500 mM represents the optimum ionic strength to shield the electrostatic repulsion and to facilitate nucleic acid hybridization [30,31]. Therefore, such a constraint limits the bioFET capabilities: indeed, the greater the ionic strength of the working solution, the smaller the Debye length within which the charges carried by the target miRNAs can be perceived [28]. One promising solution to this problem may be represented by the use of peptide nucleic acids (PNAs) as capturing probes for miRNAs. PNAs are nucleotide analogues where the negative phosphate backbones are replaced by repeated units of N-(2-amminoetil)-glycine connected by a peptide bond [32,33]. Accordingly, PNA can hybridize with complementary nucleic acids at lower ionic strengths, when compared to canonical counterparts [34,35], with a subsequent reduction of the Debye length leading to a substantial improvement of the bioFET detection capability. Therefore, this approach presents the important advantage to render accessible bioFET-based detection at physiological conditions that are usually characterized by an ionic strength of approximately 150 mM [36]. However, the absence of the negative phosphate backbones also modifies the interaction kinetics between PNA and the complementary nucleic acids; the relationship between ionic strength and PNA-miRNAs interaction on solid surfaces should be, therefore, investigated [37,38,39,40]. On such a basis, the present work has the objective of paving the way for the development of a bioFET-based biosensor for a clinically relevant microRNA at physiological-like conditions by using PNA molecules as capturing probes. Our target is microRNA 155 (miR-155), a multifunctional miRNA regulating B cell differentiation and development stages by also playing a key role in the mammalian immune system [41,42]. MiR-155 is overexpressed in various malignant tumor cells, such as hepatocellular carcinoma, breast cancer and colon cancer [43,44], whose detection deserves high interest, as a biomarker in diagnostics and prognosis [45].

Since the kinetics can be strongly modulated by the ionic strength, and on the other hand, it could have a significant impact on the bioFET response, we have preliminarily investigated the hybridization process between miR-155 and the related PNA by using surface plasmon resonance (SPR) to extract the association and dissociation rates under the experimental conditions of bioFET sensing [46,47]. We then implemented a procedure on a custom-made bioFET setup to detect miR-155 at 150 mM ionic strength. The results indicate that our label-free, PNA-bioFET system is capable of a highly specific recognition of miR-155 with a LOD in the nano-molar concentration under physiological-like conditions.

## 2. Materials and Methods

### 2.1. Materials and Reagents

Single-stranded RNA oligonucleotides with the sequence of human miR-155-5p, miR-21-5p and miR-141-5p were purchased from Metabion (Planegg, Germany) as dry pellets (see Table 1). The thiolated PNA oligomer with a complementary sequence to miR-155 was synthesized by biomers.net (Ulm, Germany) and shipped in dry form.

Oligos were purified by high-performance liquid chromatography−mass spectrometry (HPLC−MS). The pellets were resuspended in sterile 10 mM sodium phosphate buffer (NaPi, 8.1 mM Na_2_HPO_4_, 1.9 mM NaH_2_PO_4_) in aliquots with a 100 µM concentration, miRs at pH = 7.8 and the PNA at pH = 6, and then stored at 253 K. Furthermore, 6-mercapto-1-Hexanol (MCH) and other chemical reagents were purchased from Sigma-Aldrich Co., Merck KGaA, Darmstadt, Germany. For the SPR assays, customizable sensor chip Au (General Electric Healthcare, Milano, Italy) surfaces were used. For the bioFET experiments, screen-printed electrodes (DRP-220AT-U75, with a gold sensing track area of 2.0 mm^2^ purchased from METROHM Italiana Srl, Origgio, Italy) were used. The work surface and equipment were decontaminated by using RNaseZap (Ambion (Austin, TX, USA); Sigma Aldrich Co. (St. Louis, MO, USA)). The buffers were prepared using reagents from Sigma-Aldrich Co. and bi-distilled water; after being microfiltered (Sartorius, Gottingen, Germany), they were stored at 277 K and thermalized at room temperature before experiments.

### 2.2. SPR Measurements and Data Analysis

The SPR experiments were conducted at 298 K with a Biacore X100 instrument (GE Healthcare, BioSciences AB, Uppsala, Sweden). The ligand PNA was immobilized onto a sensor chip, while the analyte miR-155 was fluxed free in solution over the ligand-functionalized sensor chip. Their interaction, leading the analyte molecules to accumulate over the surface, produced changes in the refractive index of the medium, allowing us then to monitor the process in real time through the induced shift in the SPR angle.

For the immobilization, thiolated PNA solutions were incubated for 1 h with 100 mM dithiothreitol (DTT) in NaPi buffer at pH 8.0 in order to break the disulfide bond protecting the thiol (−SH) moiety; the obtained PNA-SH was eluted from NAP10 columns (GE Healthcare, Chicago, IL, USA) with working buffer (PBS, 6.84 mM Na_2_HPO_4_, 3.16 mM NaH_2_PO_4_, 126.32 mM NaCl, I = 150 mM, pH 7.2) for the removal of DTT. The sensor chip surface was cleaned with H_2_O_2_ (Merck Millipore, Darmstadt; Germany) under ultraviolet (UV) light for 30 min, according to the so-called liquid-based hydrogen-peroxide-mediated UV-photooxidation (liquid-UVPO) technique [19], washed with ultrapure water and dried with nitrogen. Once docked in the instrument, the chip was primed with running buffer (working buffer with 0.005% surfactant p20 (GE Healthcare). Using Manual Run (Biacore X100 software), an injection of 130 μL of a solution of 8 μM PNA-SH in working buffer at pH 7.2 was carried out exclusively in the measurement cell (Fc2) at a flow rate of 5 μL/min for 1080 s, while the control flow cell (Fc1) was not functionalized. Then, the Manual Run was immediately ended, and the chip was kept docked in the instrument overnight, allowing incubation at 298 K. Afterwards, in order to passivate the Au surface, both flow cells were injected with 130 μL of a solution of 1 mM MCH, in running buffer with 0.2% Ethanol, at a flow rate of 5 μL/min for 1080 s.

After a 5 h incubation, unreacted groups were finally removed from the surface by the means of 30 s pulses of 100 mM NaOH. In such a way, approximately 500 resonance units (RU) of PNA-SH were immobilized in Fc2; a rather low immobilization level was chosen to limit mass transport, rebinding and steric hindrance [47]. The interaction analyses were performed by single-cycle kinetic (SCK) and multi-cycle kinetic (MCK) assays. In SCK assays, five sequential increasing concentrations of miR-155 in running buffer (0.01, 10, 50, 175 and 750 nM) were injected over the PNA surface in both flow cells for 180 s, each followed by a 180 s dissociation step with running buffer; finally, each cycle was ended by a dissociation step of 1200 s. In MCK assays, instead, in each cycle, one of five increasingly higher concentrations (0.01−750 nM) of miR-155 was fluxed for 180 s, followed by a dissociation step of 1200 s with running buffer and by a 30 s pulse of regeneration solution (100 mM NaOH) at 30 μL/min, used to unbind analyte molecules from the PNA on the surface. After preliminary tests, to prevent mass transport [47], the fastest flow rate (30 μL/min) that allowed us to monitor the association step for the longest time was chosen. At the beginning of all assays, a bare buffer flow was used to equilibrate the surface to provide the blank response that, in combination with the control response registered from Fc1, was used to correct the sensorgrams for non-specific binding to the surface, systematic noise and instrumental drift. The experimental data were evaluated using the BiaEvaluation software 2.1 (GE Healthcare). Goodness of fits was estimated from the residual plots, χ^2^ value and U value, the latter estimating the uniqueness of the calculated parameters (not significantly correlated for U < 15) [48]. The measurements were conducted in triplicate.

### 2.3. Sensing Area Functionalization of bioFET Electrodes

The gold sensing area of the electrodes was cleaned and activated using the liquid-UVPO technique [49]. The biofunctionalization procedure of active electrodes by PNA exploited the formation of a self-assembled monolayer (SAM) through the thioester bond generated between the terminal thiol of PNA probes and the gold surface of the electrodes [50]. It consisted of two steps separated by rinsing the gold surface with filtered (0.2 µm filtering membrane pore size) deionized water and drying by pure nitrogen. In the first step, the gold sensing area was incubated with 12 µL of a mixed solution of PBS (6.84 mM Na_2_HPO_4_, 3.16 mM NaH_2_PO_4_, 126.32 mM NaCl, I = 150 mM, pH 7.2) containing 350 µM MCH and 15 µM PNA for 3 h at room temperature. The role of MCH molecules in the first co-immobilization step was to distance the PNA probes from each other, thus, making them more accessible for binding with miR-155 [51,52,53]. In the second step, the electrodes were incubated with 12 µL of PBS containing 1mM MCH for one hour at room temperature, to block the unreacted site of the gold sensing area. For what concerns the control electrodes, only the second immobilization step was implemented. The sensor surface of the electrodes was then rinsed as described above and the sensors were used immediately.

### 2.4. bioFET Setup, Measurements and Data Analysis

Functionalized electrodes were inserted into a printed circuit board (PCB) with an integrated commercial zero-threshold n-type metal oxide semiconductor FET (mosFET; ALD110900A from Advanced Linear Devices Inc., Sunnyvale, CA, USA). The electrodes were then horizontally inserted into a commercial fluidic cell (METROHM Italiana Srl) characterized by a conical opening able to accommodate the functionalized sensing area of electrode together with the commercial bulky Ag/AgCl reference electrode (DriRef-2, World Precision Instruments Ltd., Hitchin, UK). The use of the fluidic cell provides a high stability to the system. The conical opening of the fluidic cell also allowed target injections to be performed in a controlled manner and in proximity to the functionalized electrode sensing area. The experimental measurements were carried out by a Keithley 2636B (TeKtronix, Beaverton, OR, USA), with a sensitivity in the fA range for current measurements and µV range for potential ones. Two source-mater units (SMUs) of the Keithley apparatus were used. The SMU1 applied the voltage (V_ds_) between the drain and source electrodes, which was kept constant at 100 mV for all experiments, and measured the corresponding current flow (I_ds_). The SMU2 applied a potential bias (V_ref_) to the reference electrode in the solution. Images of the bioFET setup are shown in Figure S1 in Supplementary Materials.

The real-time assays were performed at constant Vds (100 mV) and Vref (450 mV) and the Ids current was monitored over-time. First, 15 µL volumes at increasing concentrations of miR-155 were added by pipetting into the conical opening of the fluidic cell (the same working buffer was used to avoid current variation as a result of the change in pH or ionic strength in the solution [54]). Biosensing experiments were carried out in NaPi 150 mM pH = 7.2 as working buffer (working volume of 200 µL).

Prior to the real biosensing analyses, the real-time current was registered until it reached a plateau sufficient to ease later biosensing tests and the related signal evaluation (see Figure S2 in Supplementary Materials) [55]. In this regard, the injections were started when an average current variation of less than 0.01 μA over 200 s was detected. The current value after stabilization was normalized to the current variation according to the formula (I_ds_ − I_0_)/I_0_ = ∆I/I_0_, where I_ds_ stands for the real-time recorded current, while I_0_ is the current value obtained just after stabilization [56]. Furthermore, five single injections of working buffer were performed on active electrodes to obtain the blank signal required for the calculation of the LOD of our system. LOD, defined by IUPAC as the smallest measure that can be reasonably detected for a given analytical procedure [56], was calculated from the calibration curve, using five times the standard deviation of the blank, according to the procedure in refs. [57,58] (see also ref. [59]). Finally, the specificity tests were carried out in the same conditions described above but substituting mirR-155 with noncomplementary miRNAs. All biosensing experiments, as well as the control tests, were repeated three times. Data analyses, plotting and fitting were performed using the Microcalc OriginPro 8.5 software product (Origin Corporation, Northampton, MA, USA).

## 3. Results and Discussion

### 3.1. SPR Investigation

**Figure 2:**
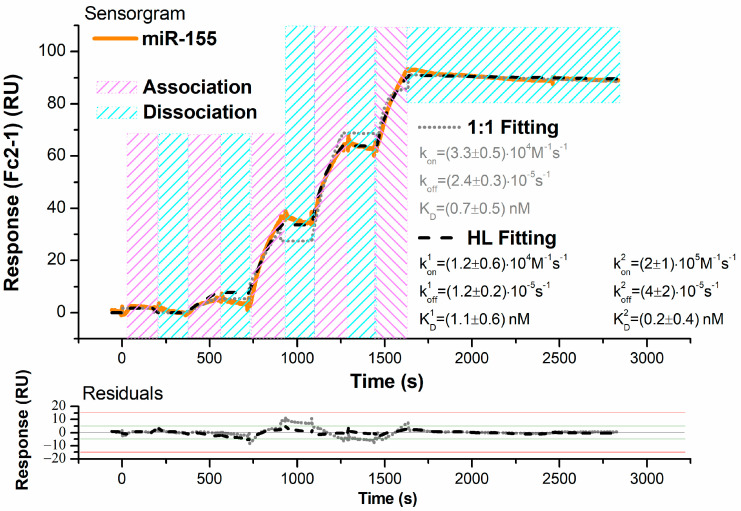
Top: SPR sensorgram (solid orange curve) of a representative SCK assay performed at 298 K upon injection of five increasing concentrations (0.01–750 nM) of miR-155 in running buffer over the PNA-functionalized sensor chip surface. Global fit of the sensorgram was performed according to a 1:1 reversible bimolecular binding model (dotted grey curve; scoring χ^2^ = 8.88 RU^2^) and to the HL binding model (dashed black curve; scoring χ^2^ = 2.54 RU^2^); the results of the fittings are also shown. Bottom: plot of the fitting residuals.

The kinetics of PNA/miR-155 hybridization in physiological-like conditions were investigated by SPR kinetic assays. Indeed, the SPR set up, although based on a continuous-flow microfluidic approach, allows us to up-close mimic the conditions occurring in biosensing experiments, in which the molecular probe, immobilized on the gate surface, interacts with the target specimen free in solution. The gold-coated surfaces of the SPR sensor chips were functionalized with PNA molecules (see Section 2, and subsequently submitted to the flow of miR-155 in running buffer (NaPi, I = 150 mM, pH 7.2). Figure 2 shows the monitored response over time (sensorgram) of a representative SCK assay, in which solutions of five increasing concentrations (0.01, 10, 50, 175, 750 nM) of miR-155 were fluxed over a PNA-immobilized chip, separated by bare buffer injections of the same volume and followed by a final, prolonged (1200 s) flow of bare buffer.

**Figure 3:**
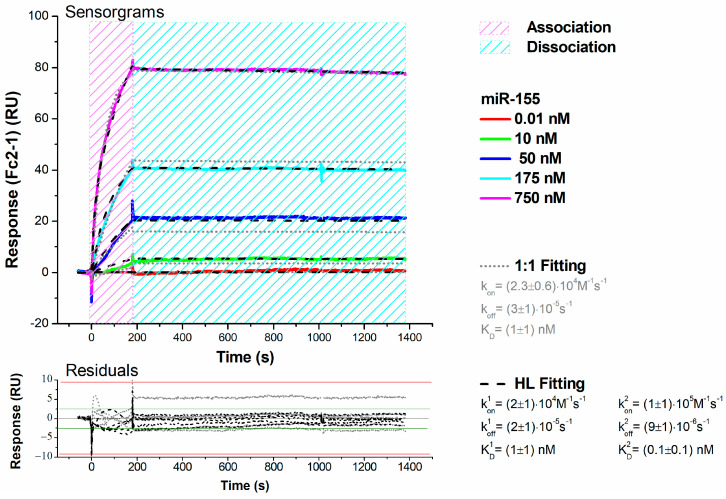
Top: SPR sensorgrams (solid-colored curves) of the MCK assay performed at 298 K upon injection of five increasing concentrations (0.01−750 nM) of miR-155 in running buffer over the PNA-functionalized substrate. Global fit of the sensorgrams was performed according to the 1:1 binding model (dotted grey curves; scoring χ^2^ = 7.84 RU^2^) and to the HL binding model (dashed black curves; scoring χ^2^ = 4.05 RU^2^); the results of the fittings are also shown. Bottom: plot of the fitting residuals.

During miR-155 injections, the registered response presents a significant rise, signaling that more and more miR-155 molecules carried in the continuous flow of buffer are being held close to the surface by the specific interaction with PNA (association phase). Moreover, these ascending curves do not level out within the analyte injection period (180 s), hinting at a rather slow dissociation rate. As the analyte carrying flow is substituted by buffer alone to promote removal of miR-155 from the surface (dissociation phase), a comparatively much smaller fall of the signal can be observed: the response decreases only by 5% on average, even after a long waiting time (1200 s). These results indicate a quite strong and long-lived interaction between the partners. First, we have fitted the obtained sensorgram using the 1:1 Langmuir binding model, which assumes a simple reversible biomolecular reaction [60]. By obtaining a score of χ^2^ = 8.88, we found a value of (3.3 ± 0.5) × 10^4^ M^−1^ s^−1^ for the association rate constant (k_on_) and of (2.4 ± 0.3) × 10^−5^ s^−1^ for the dissociation rate constant (k_off_), leading to an equilibrium dissociation constant (K_D_ = k_off_/k_on_) of 0.7 ± 0.5 nM. As shown in Figure 2, the 1:1 fitting curve shows slight deviations from the experimental data. Similar deviations from the Langmuir trend have been observed for the hybridization kinetics of oligonucleotides immobilized on copolymer-coated glasses, and they have been ascribed to some inhomogeneity in the arrangement of molecules on the surface, giving rise to an accumulation of charges onto the surface and then to steric hindrance [61]. Accordingly, the sensorgrams were analyzed through the heterogeneous ligand (HL) binding model, which considers some heterogeneity in the layer of surface-attached molecules and describes the binding curve as the sum of two main independent sub-populations. HL fitting scores a better χ^2^ = 2.54, with uniformly distributed residuals of the small entity (see Figure 2) and identifies two equal-weight contributions characterized by: k_on_^1^ = (1.2 ± 0.6) × 10^4^ M^−1^ s^−1^ and k_off_^1^ = (1.2 ± 0.2) × 10^−5^ s^−1^, k_on_^2^ = (2 ± 1) × 10^5^ M^−1^ s^−1^ and k_off_^2^ = (4 ± 2) × 10^−5^ s^−1^; the corresponding equilibrium dissociation constant being K_D_^1^ = 1.1 ± 0.6 nM and K_D_^2^ = 0.2 ± 0.4 nM. Notably, the values of the association and dissociation rate constants extracted by the two applied models are quite similar; this suggests that PNA/miR-155 hybridization can be suitably described by a 1:1 reversible reaction, while the heterogeneity in the PNA layer, possibly causing different accessibility to the surface binding sites, does not substantially alter the interaction kinetics. To further verify these results, kinetic assays were also performed using the MCK approach, in which a regeneration step is carried out after each miR-155 injection (see Section 2). Since only minimum dissociation can be obtained by the fluxing buffer alone, 30 s long pulses of 100 mM NaOH at 30 µL/min were used to remove from the surface miR-155 molecules hybridized to the PNA before injecting the next concentration of miR-155. Such an approach, under the assumption that the repeated exposure to the regeneration solution does not alter the PNA layer, also allows us to better estimate the value of k_off_, as all the analyte injections are followed by prolonged (1200 s) dissociation steps. In Figure 3, the sensorgrams of a representative MCK assay show progressively higher signals as the concentration of the injected miR-155 solutions increases from 0.01 to 750 nM.

The trend of the sensorgrams is similar to that observed with the SCK approach, with response curves rising without reaching a plateau within the association phases and not significantly decreasing during the dissociation steps. A fitting of the MCK data by the 1:1 Langmuir binding model, with χ^2^ = 7.84, has provided: k_on_ = (2.3 ± 0.6) × 10^4^ M^−1^ s^−1^, k_off_ = (3 ± 1) × 10^−5^ s^−1^ and K_D_ = 1 ± 1 nM. Conversely, the HL fitting, with χ^2^ = 4.05, identifies two contributions: the main described by k_on_^1^ = (2 ± 1) × 10^4^ M^−1^ s^−1^, k_off_^1^ = (2 ± 1) × 10^−5^ s^−1^, K_D_^1^ = 1 ± 1 nM and the minor by k_on_^2^ = (1 ± 1) × 10^5^ M^−1^ s^−1^, k_off_^2^ = (9 ± 1) × 10^−6^ s^−1^, K_D_^2^ = 0.1 ± 0.1 nM. The MCK assay has provided association rates comparable to the ones obtained by the SCK method and even slower dissociation rates (at the detection limit), confirming a quite strong and long-lasting interaction between the partners. Accordingly, at the physiological ionic strength, the association between immobilized PNA and free miR-155 molecules is an efficient process, with hybridization occurring within rather short times. On the other hand, the complex dissociation rates are characterized by a very long lifetime, indicating that the formed duplex is quite stable. Notably, the found results indicate a more stable interaction in comparison to that observed between miR-155 and its complementary RNA strands (antimiR-155) in similar measurement conditions [62]. Finally, the resulting K_D_ values denote a high affinity that falls within the range observed for duplex formation of oligonucleotides free in solution [63]. Furthermore, the evidence that, even in the SCK approach, the binding processes better describe the kinetics supports some heterogeneity in the binding process.

**Figure 4:**
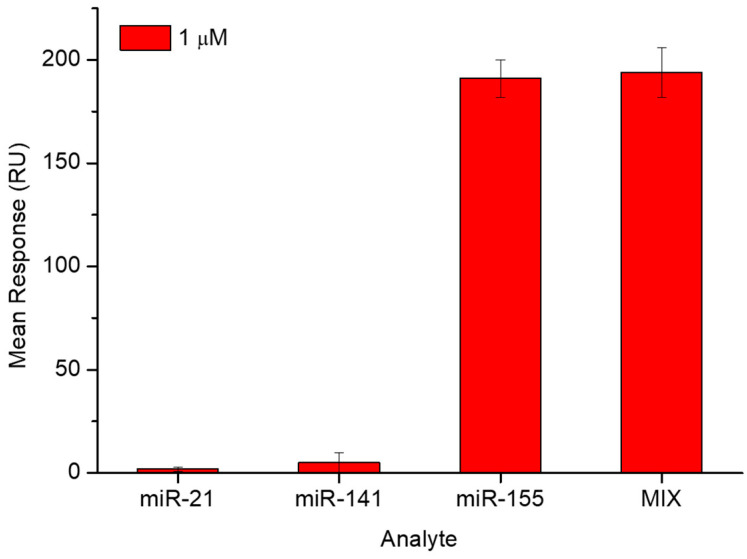
Comparison of SPR responses of the same PNA-functionalized sensor chip against the target (miR-155, at 1 mM), two non-complementary miRNA strands (miR-141 and miR-21, both at 1 mM) and a mixture (MIX) of all three miRNAs, each one at the concentration of 1 µM. All measurements were performed in running buffer.

To evaluate the specificity of the PNA-functionalized surface for the detection of miR-155, control SPR experiments with nontarget miRNAs were performed. A sensor chip immobilized with PNA was exposed to the flow of 1µM solutions in running buffer of miR-21; miR-141, miR-155 and a mix of all three miRNAs. For each case, the test was repeated three times, with the surface being regenerated after each analyte injection. A comparison of all the collected SPR responses is shown in Figure 4.

A substantially negligible signal was obtained for miR-21, and the response of miR-141 was 90% lower than that observed with miR-155. Comparing the miR-21 and miR-141 groups with the MIX group by *t*-test, they differed at a significant level of 1%; while the signal of the miR-155 group and that of the MIX group is substantially the same at a significance level of 10%. The used non-complementary miRNA strands did not generate a significant system response; with this supporting the high specificity of the PNA towards miR-155.

### 3.2. Biosensing Analyses by a bioFET Setup

SPR results indicate that in hundreds of seconds, the interaction between the partners has largely occurred. Although SPR results have been obtained in microfluidic conditions while bioFET experiments should be performed in static fluid conditions, they provide some preliminary information about the temporal window helping to define real time bioFET experiments. The gold sensing area of the electrodes (representing the functional extended gate of the bioFET system) was functionalized with PNA molecules. In parallel, control electrodes without probes were prepared (for more details see Section 2). The gold electrodes were subsequently connected to the gate of bioFET and inserted into the fluidic cell, which was, in turn, filled with a working buffer solution identical to that used for SPR assays. In all the experiments, prior to real biosensing analyses, the real-time current was registered until it reached a plateau sufficient to ease the later biosensing tests and the related signal evaluation [64]. In this regard, it was decided to start the injections at an average current variation of less than 0.01 μA over 200 s. Notably, electrodes reached the stability at slightly different times; with this being likely due to small fabrication differences between electrodes and to the heterogeneity of the functionalized layers. However, in almost all the cases, the stability was reached within a waiting time from 1000 to 2000 s. After current stabilization, multiple injections of solutions containing increasing concentrations of miR-155 were performed, at 100 s time intervals, on both active and control electrodes, by continuously recording the current.

**Figure 5:**
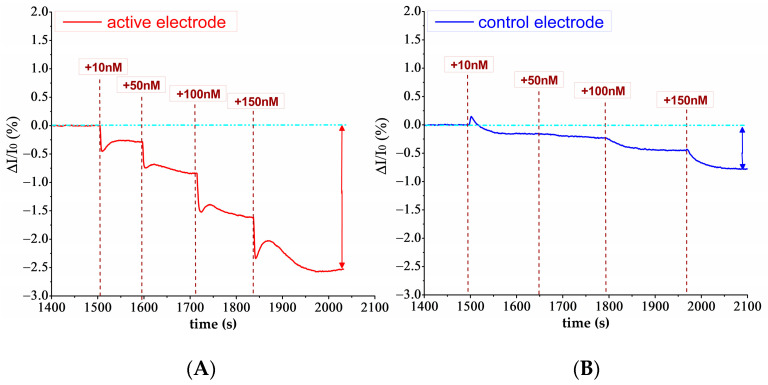
Real time biosensing analysis. Sequential injections of miR-155 at increasing concentrations (10 nM, 50 nM, 100 nM and 200 nM) on PNA-functionalized active electrode (**A**) and on MCH control electrode (**B**).

Figure 5A,B show the real-time responses of representative biosensing assays performed on both the active and control electrodes, respectively, by adding successive solutions of miR-155 at 10, 50, 100 and 150 nM concentrations.

Upon adding a miR-155 containing solution, a rapid current drop occurs, followed by a rise that reaches a sort of equilibrium, indicating that the hybridization of miR-155 molecules with PNA probes has occurred. Such a behavior finds a correspondence with the SPR results and indicates that immobilized PNA probes efficiently capture the target miR-155 in solution within a relatively short time (about 100 s).

To compare the injection effects occurring on different electrodes, a normalization to the current variation was performed (see Section 2). As shown in Figure 5A, drops of I_ds_ are detected in all the cases upon adding solutions containing the target. Such a behavior is consistent with the negative charges that are released to the gate by the captured miR-155 molecules at pH = 7.2 [64] and with the fact that our bioFET uses a n-type transistor in which the main electric carriers are electrons. Indeed, in these operative conditions, the negative charges provided by the miR-155 molecules move away the electrons from the conductive channel, connecting the drain and source electrodes and cause a decrease in the recoded I_ds_. Furthermore, progressively higher current drops are detected as far as the concentration of the target in the fluidic cell increases.

Injections of miR-155 carried out on the control electrode (see Figure 5B) have yielded a much lower decrease in current. These I_ds_ variations could be attributed to random non-specific adsorption, which may persist even after passivation of the gold electrode surface [65]. With the aim of eliminating contributions given by such non-specific interactions, we have subtracted from the current variations ((∆I/I_0(A)_) of the active electrodes the corresponding current detected for the control electrodes (∆I/I_0(C)_), giving as a final signal (∆I/I_0(A)_ − ∆I/I_0(C)_).

**Figure 6:**
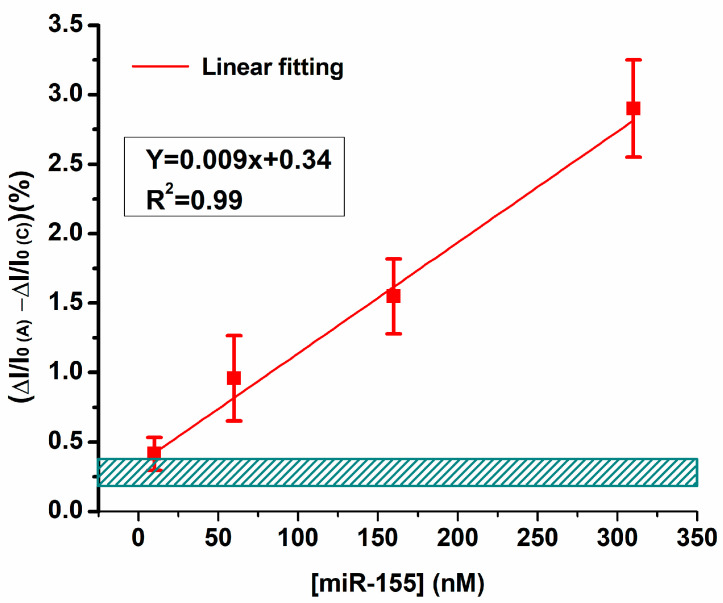
BioFET calibration curve obtained by plotting the normalized current variation vs. miR-155 concentration. Red squares indicate the current variation observed by injecting miR-155 on active PNA electrode ∆I/I_0(A)_ minus the variations associated with the same injections on control electrodes (∆I/I_0(C)_). The barred box at the bottom represents the signal associated with buffer injections plus three times the relative standard deviation.

Notably, the very long dissociation time evaluated by SPR allows us to approximate that the almost totality of the PNA-bound miR-155 molecules will not dissociate by the end of the measurement (i.e., within 600 s after the first injection). Indeed, the final I_ds_ response corresponds to the total miR-155 concentration (as obtained by cumulating the different injections) [64]. Accordingly, the effective concentrations of miR-155 in the fluidic cell at each step are approximately 10, 60, 160 and 310 nM. The obtained signal variations were plotted vs. the miR-155 concentration to construct the calibration plot, as shown in Figure 6.

The PNA-bioFET system response is characterized by a good linearity, as witnessed by the R^2^ = 0.988 value. Globally, these results indicate that bioFET allows a robust and sensitive detection of miR-155 at 150 mM ionic strength, in agreement with the kinetic characteristics found in SPR (fast association, slow dissociation and high affinity interaction). Furthermore, from the signal obtained from five single injections of the working buffer performed on active electrodes, we have estimated an LOD of approximately (5 ± 2) nM (see also Section 2). Such a value falls in the range found for other electrochemical biosensors for miR-155 [66] and different microRNAs of clinical interest [10,38,39,67]. The response of the system to injections of 1 nM and 5 nM is shown in Supplementary Materials (see Figure S3).

We would also like to remark that the use of PNA has allowed us to improve the detection level in comparison to the use of the complementary strand as a detecting probe (see Figure S4 in Supplementary Materials).

**Figure 7:**
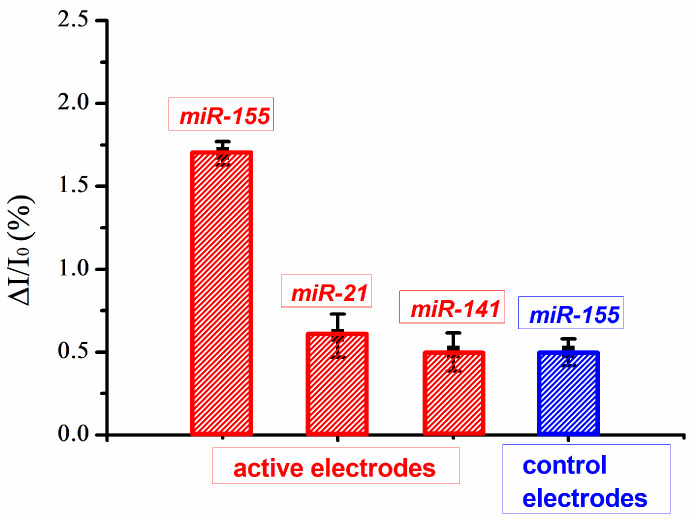
Normalized current variation associated with miR-155, miR-21 and miR-141 injections on active electrodes (red) and associated with miR-155 injections on the control electrode (blue). For all the cases, a concentration of 150 nM was used.

Finally, the specificity of the bioFET setup was evaluated by adding the same non-complementary miRNAs (miR-21 and miR-141), as performed in SPR testing. For this purpose, sequential injections of miR-21, miR-141 and miR-155 at concentration of 150 nM were carried out on the active electrodes. The collected PNA-bioFET responses are shown in Figure 7.

Non-complementary miRNA injections have yielded a much lower current variation, which is indeed comparable to that occurring when miR-155 is injected in non PNA-functionalized electrodes (control electrodes in Figure 7, blue box). The residual activity for nonspecific miRNAs could be explained in terms of non-specific adsorption, in some respect, being favored by the static nature of electrode wetting in our bioFET system [64].

## 4. Conclusions

PNA molecules were revealed to be extremely promising capture probes for miR-155, allowing us to reach an efficient hybridization even at relatively low ionic strengths. The investigation by using SPR of the interaction kinetics between PNA molecules immobilized on a gold-coated surface with miR-155 in fluxed solution at an ionic strength of 150 mM has provided association and dissociation rates of k_on_ = 2·10^4^ M^−1^ s^−1^ and k_off_ = 2·10^−5^ s^−1^, respectively, and an affinity of approximately 10^9^ M^−1^. These results, on one hand, indicate that PNA can hybridize miR-155 even at the ionic strengths mimicking the physiological conditions and, on the other, that they may represent an optimal capture probe for bioFET experiments. BioFET measurements performed by successive injections at progressively higher concentrations of miR-155 have allowed us to extract a biosensing calibration plot characterized by a very good linearity in the 10–150 nM miR-155 concentration range and showing an LOD of approximately 5 nM. Control bioFET experiments with other miRNAs, such as miR-21 and miR-141, have shown that PNA is quite specific in capturing miR-155. The remarkable performance reached in the present study in terms of rapidity, specificity and absence of labels indicates that our bioFET sensing approach could be very promising to be translated in clinical diagnostics in physiological fluids.

## Figures and Tables

**Table 1:** Sequences of oligonucleotides used in this work (the complementary portions of PNA and miR-155 involved in duplex formation are highlighted in yellow).

Oligonucleotides	Sequences
PNA	5′-aac ccc tat cat tat tag cat taa-3′
miR-155	5′-uaa ugc uaa ucg uga uag ggg-3′
miR-21	5′-uag cuu auc aga cug aug uug-3′
miR-141	5′-uaa cac ugu cug gua aag aug-3′

## Data Availability

Data are contained within the article.

## Supplementary Materials

The following supporting information can be downloaded at: https://www.mdpi.com/article/10.3390/bios14020079/s1, Figure S1: Images of the experimental bioFET setup used for detection of miR-155. (A) Keithley apparatus used to apply the selected potentials and to record the current for bio-sensing experiments; (B) zoom of the conic fluid cell during the injection; Figure S2: A representative stabilization curve of functionalized electrodes obtained before performing injections of target; Figure S3: Real time biosensing analysis. Injections of miR-155 at the concentrations of (A) 1nM, and (B) 5nM, on PNA-functionalized active electrode; Figure S4: Representative biosensing assay using antisense RNA (antiMir-155) as probe in experimental conditions comparable to the biosensing experiments using PNA as probe.
