# Supplementary material for: Detection of miR-155 Using Peptide Nucleic Acid at Physiological-like Conditions by Surface Plasmon Resonance and Bio-Field Effect Transistor
Source: Biosensors (Basel). 2024 Feb 1;14(2):79. doi: 10.3390/bios14020079 (PMC10887097; doi:10.3390/bios14020079)
Supplement: Supplementary file 1 [file biosensors-14-00079-s001.zip › biosensors-2812181-supplementary.pdf]

biosensors

Supplementary Materials

Detection of miR-155 Using Peptide Nucleid Acid at Physiological-like Conditions by Surface Plasmon Resonance and Bio-Field Effect Transistor

Francesco Lavecchia di Tocco, Valentina Botti, Salvatore Cannistraro and Anna Rita Bizzarri *

Biophysics and Nanoscience Centre, DEB, Università della Tuscia, Largo dell'Università, 01100 Viterbo, Italy; francesco.ditocco@unitus.it (F.L.d.T.); valentina.botti@unitus.it (V.B.); cannistr@unitus.it (S.C.)
* Correspondence: bizzarri@unitus.it

Images of the experimental setup are shown in Figure S1.

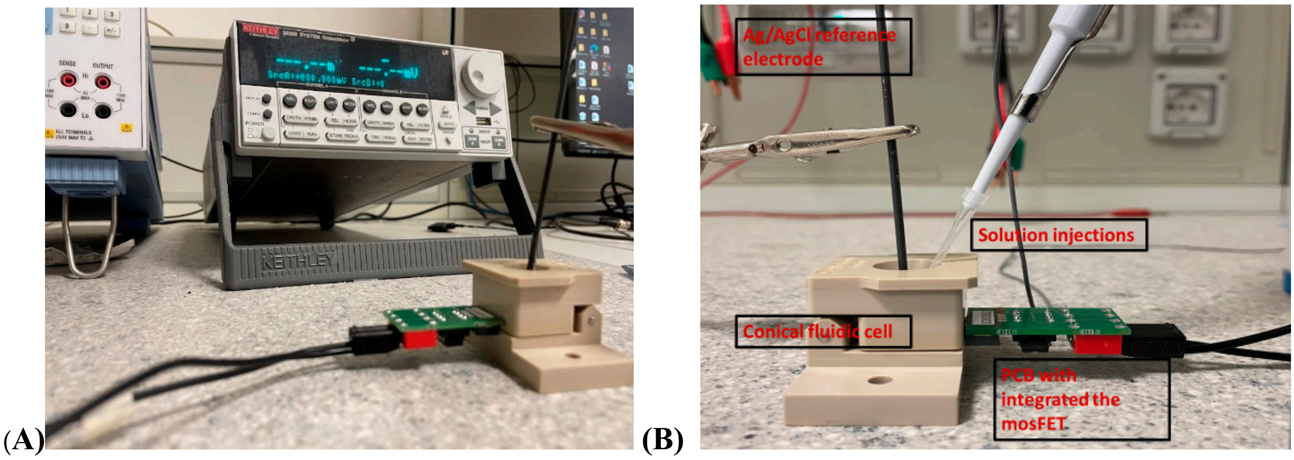

Figure S1. Images of the experimental bioFET setup used for detection of miR-155. (A) Keithley apparatus used to apply the selected potentials and to record the current for bio-sensing experiments; (B) zoom of the conic fluid cell during the injection.

A representative Ids vs. time stabilization curve of functionalized electrodes is shown in Figure S2. The curve shows an initial increase in the order of µA with an approximately exponential trend before reaching a stable value. In almost all the cases, stability is reached between 1000 and 2000 seconds and the Ids value reached at equilibrium (I_0) ranging from 40 to 50 uA for functionalized electrodes. Such differences could be ascribed to small constructive differences between electrodes and to the heterogeneity of functionalized layer.

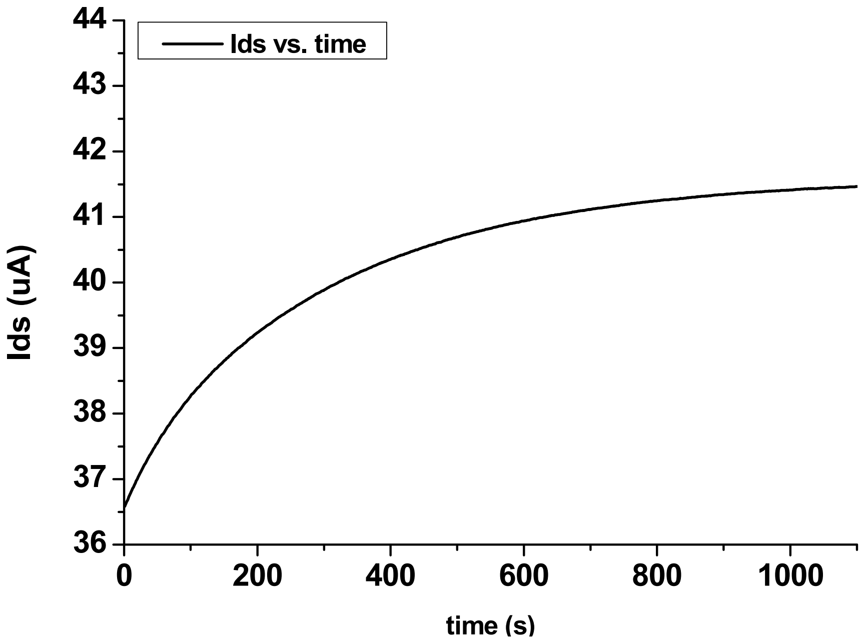

Figure S2. A representative stabilization curve of functionalized electrodes obtained before performing injections of target.

Figure S3 show the real-time responses of representative biosensing assays performed on electrode functionalized with PNA with injection of 1 nM and 5nM.

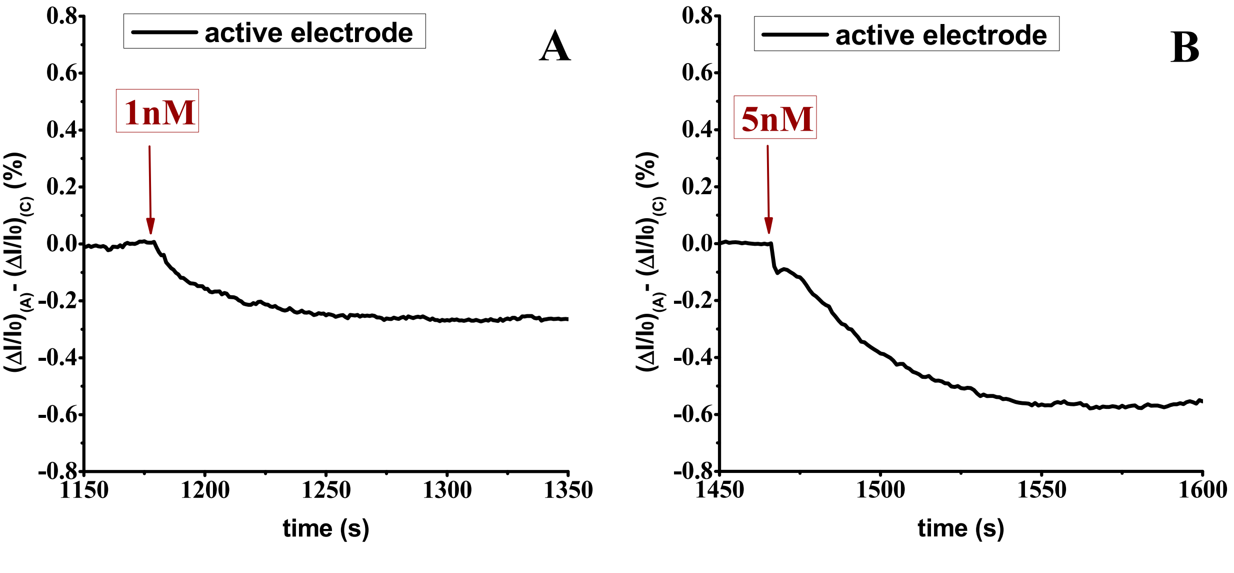

Figure S3. Real time biosensing analysis. Injections of miR-155 at the concentrations of A) 1nM, and B) 5nM, on PNA-functionalized active electrode.

Figure S4 shows the real-time responses of representative biosensing assays performed on the active electrode functionalized with anti miR-155 (aaa aaa aac ccc uau cac gau uag cau uaa-3′ tagged with the ThiolC6 linker group) complementary to miR-155, by adding successive solutions of miR-155 at 10, 50, 100, 150 nM concentrations. The electrode has been prepared by following the same procedure used for PNA. Upon adding a miR-155 containing solution, a significant current drop occurs only at concentrations higher than 150 nM. Additionally, the current drop is lower in comparison to that detected using as probe PNA at the same miR-155 concentration (see Figure 5).

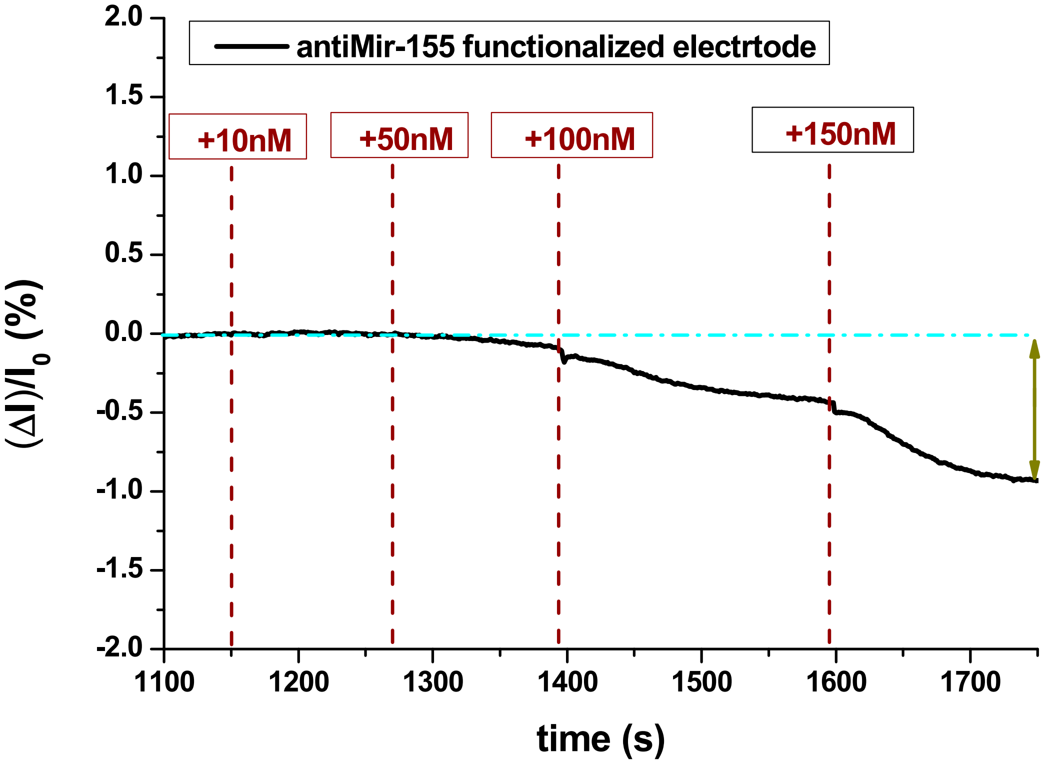

Figure S4. Representative biosensing assay using antisense RNA (antiMir-155) as probe in experimental conditions comparable to the biosensing experiments using PNA as probe.
